# Joint Coherent/Non-Coherent Detection for Distributed Massive MIMO: Enabling Cooperation Under Mixed Channel State Information

**DOI:** 10.3390/s25216800

**Published:** 2025-11-06

**Authors:** Supuni Gunasekara, Peter Smith, Margreta Kuijper, Rajitha Senanayake

**Affiliations:** 1 Department of Electrical and Electronic Engineering, University of Melbourne, Parkville, VIC 3010, Australia; 2 School of Mathematics and Statistics, Victoria University of Wellington, Wellington 6140, New Zealand

**Keywords:** cell-free networks, coherent detection, cooperative communications, distributed antenna systems, massive MIMO, non-coherent detection

## Abstract

Beyond-5G wireless systems increasingly rely on distributed massive multiple-input multiple-output (MIMO) architectures to achieve high spectral efficiency, low latency, and wide coverage. A key challenge in such networks is that cooperating base stations (BSs) often possess different levels of channel state information (CSI) due to fronthaul constraints, user mobility, or hardware limitation. In this paper, we propose two novel detectors that enable cooperation between BSs with differing CSI availability. In this setup, some BSs have access to instantaneous CSI, while others only have long-term channel information. The proposed detectors—termed the coherent/non-coherent (CNC) detector and the differential CNC detector—integrate coherent and non-coherent approaches to signal detection. This framework allows BSs with only long-term information to actively contribute to the detection process, while leveraging instantaneous CSI where available. This approach enables the system to integrate the advantages of non-coherent detection with the precision of coherent processing, improving overall performance without requiring full CSI at all cooperating BSs. We formulate the detectors based on the maximum likelihood (ML) criterion and derive analytical expressions for their pairwise block error probabilities under Rayleigh fading channels. Leveraging the pairwise block error probability expression for the CNC detector, we derive a tight upper bound on the average block error probability. Numerical results show that the CNC and differential CNC detectors outperform their respective single-BS baseline-coherent ML and non-coherent differential detection. Moreover, both detectors demonstrate strong resilience to mid-to-high range correlation at the BS antennas.

## 1. Introduction

Massive multiple-input multiple-output (MIMO) systems are a cornerstone of modern wireless communication systems, offering significant improvements in spectral efficiency, reliability, and network capacity [1,2]. By employing a large number of antennas at the base station (BS), massive MIMO systems are able to provide substantial improvements in data rates and serve multiple users simultaneously, leveraging spatial multiplexing and diversity gains.

Traditional approaches to user signal detection in massive MIMO systems can be broadly classified into two categories: coherent detection [3,4,5,6,7,8,9], which depends on instantaneous channel state information (CSI), and non-coherent detection [10,11,12,13,14,15,16,17,18,19], which operates using long-term channel information. Using pilot-based mechanisms to acquire channel estimates, coherent detection offers excellent performance under ideal circumstances [5]. Several works [3,4,5] have examined the performance of such systems under centralized and decentralized architectures. However, these systems are susceptible to channel variations and require regular CSI updates, which impose substantial overhead in terms of signaling and computational complexity. Recent studies have proposed scalable and practical solutions to mitigate these challenges. For instance, ref. [6] introduces subset minimum mean squared error receivers to reduce computational burden, while [7,8,9] propose partial joint processing and clustering architectures that reduce network overhead while maintaining performance.

In contrast, non-coherent detection relies on long-term channel information, which often results in a substantially inferior performance compared to coherent detection [14]. However, BSs operating non-coherently benefit from low computational complexity, power consumption [20] and reduced overhead associated with CSI updates. Several non-coherent detection strategies have been proposed for use in massive MIMO systems. These systems, mainly utilize approaches based on differential detection [10,11,12,13] and energy detection [14,15,16,17]. Differential detection strategies encode information in the phase difference across time without requiring CSI. For example, refs. [10,12] explore differential phase shift keying schemes under various fading environments, while ref. [11] studies non-coherent detection from a system-level perspective. Works such as [13,18,19] examine the effect of spatial correlation and distributed implementations, providing insight into practical performance in large-scale arrays. Energy-based non-coherent detection relies on received signal strength rather than phase information. While [15,16,17] examine energy-based modulation schemes in various setups, ref. [14] proposes constellation design strategies for energy based non-coherent massive MIMO systems. In addition to these traditional paradigms, several works have explored joint channel estimation and signal detection [21,22,23], where the channel state and transmitted data symbols are estimated simultaneously-circumventing the need for dedicated pilot signaling.

In non-cooperative cellular networks, users located near the cell edge suffer from significantly lower signal-to-interference-noise ratios (SINR) compared to those closer to the center. This degradation occurs for two main reasons: as users move farther from the BS, the received signal power diminishes rapidly with the propagation distance [24]. Additionally, because each user is exclusively served by a single BS, signals from users in neighboring cells are treated as interference. The combined impact of these factors leads to a notable reduction in SINR at the cell edge, which in turn causes substantial variations in data rates across the cell. These disparities present challenges for maintaining the high data rates required by modern mobile networks. Distributed massive MIMO systems have been proposed as a solution, with coherent and non-coherent approaches addressing the problem in different ways. Here, geographically distributed BSs equipped with massive MIMO arrays are interconnected via fronthaul links to a central processing unit (CPU). Note that, in this work, the term CPU refers to the central processing unit of the network, i.e., the entity where signals from distributed BSs are aggregated via fronthaul links and jointly processed. This setup enables the CPU to detect signals from all users within the cooperating cells, effectively managing network resources and improving overall service quality. Coherent cooperative systems can combat interference by exploiting the CSI of traditionally interfering users, thus improving the SINR [25]. On the other hand, non-coherent cooperative systems enhance performance by accumulating more signal power from the additional antennas provided through cooperation, despite not relying on real-time CSI [18].

### 1.1. Motivation

Conventionally, cooperation in wireless systems has been limited to BSs utilizing the same type of processing: either coherent-to-coherent [3,9,26] or non-coherent-to-non-coherent [18,19] coordination. The underlying idea is that cooperation is most effective when the detection capabilities of the BSs are aligned. Nonetheless, there may be instances where enhancing system performance is crucial, yet locating BSs that share the same processing techniques for cooperation can be problematic. In such cases, involving BSs with alternative processing techniques could help maintain the required level of error performance.

In the literature, coherent and non-coherent detection methods are typically applied in isolation, with systems rarely utilizing both approaches simultaneously. A hybrid demodulation approach that attempts to combine the benefits of both techniques was introduced in [27]. This method uses differentially-encoded data streams instead of traditional pilot symbols, and the data is demodulated using non-coherent detection at the BS to support channel estimation. However, this system still applies coherent and non-coherent detection in two separate layers rather than truly integrating them. Moreover, these methods perform well when the system operates under uniform channel conditions or when all cooperating BSs can provide coherent channel estimates. However, they do not address the increasingly common situation in modern wireless systems where some BSs may only be able to provide non-coherent signal observations due to hardware limitations, synchronization issues, fronthaul constraints, or high user mobility. More importantly, even if coherent CSI could be acquired at more BSs, doing so involves significant signaling and computational overhead. Therefore, limiting the number of BSs that acquire instantaneous CSI is not only realistic but also a strategy to reduce system complexity and improve scalability.

Building on these insights, in this paper, we propose two novel detectors that integrate coherent and non-coherent detection schemes into a unified approach for user data detection, enabling both methods to work simultaneously. In contrast to conventional detection approaches restricted to a single mode of cooperation, the proposed method provides a fresh perspective by jointly exploiting both coherent and non-coherent modes to enhance user performance. As discussed earlier, coherent detection leverages phase-aligned signals using known CSI, while non-coherent detection relies on energy or amplitude without requiring precise phase knowledge. Although fundamentally different, both types of information contribute to the likelihood of each symbol hypothesis. The coherent component provides a phase-sensitive term, while the non-coherent component contributes a phase-invariant, energy-based term. The joint likelihood function naturally integrates both, making the joint detection approach not only feasible but also highly relevant for next-generation wireless networks.

### 1.2. Contribution

We consider a distributed massive MIMO network where BSs cooperate to serve a single-antenna user (A natural extension is the multi-user case; however, this lies beyond the present scope as it would introduce inter-user interference and scheduling aspects.). While some BSs possess instantaneous CSI, another set of BSs contribute to the cooperative detection process by relying solely on long-term channel information. We use the terms *coherent* and *non-coherent* to categorize BSs according to their access to user channel information, distinguishing between those with instantaneous user channel information and those with only long-term channel information. The coherent and non-coherent BSs are linked to a CPU through a fronthaul network. The user channel information (be it instantaneous or long-term) and the user signals received at these BSs are transmitted to the CPU, where user signals are jointly detected. Our objective is to evaluate the potential performance improvements when a user is supported by additional BSs that operate differently from their primary BSs. It should be noted that the term primary is adopted solely for explanatory purposes to refer to the BSs initially serving the user. In practice, there is no distinction between cooperating BSs beyond whether they operate in coherent or non-coherent mode. We study this in two complementary perspectives as discussed below.

We further consider a scenario where the user is initially served by a primary BS-operating either coherently or non-coherently, depending on its processing capability. If this link’s quality degrades due to fading, mobility, or interference, neighboring BSs may assist opportunistically in whichever mode their CSI availability allows.

- **Coherent primary BSs with non-coherent assistance**: In the first scenario, we consider a user primarily served by a set of coherent BSs, without differential encoding. As the user signals are being detected coherently, the instantaneous user channels from the user to this set of BSs are known. Some other BSs, while able to contribute to the detection of user signals, lack the capability to access instantaneous user channel information and are restricted to using long-term channel information. We introduce one or more such non-coherent BSs to assist in the detection process, allowing the system to leverage non-coherent benefits in conjunction with coherent detection. Given that this novel detector leverages both coherent and non-coherent approaches for data detection, we term this as the *coherent/non-coherent detector (CNC detector)*. We propose that this method could be implemented as an inter-tier cooperative scheme for heterogeneous networks [28,29]. For example a massive MIMO BS in the coverage tier can provide aid in detection non-coherently to a set of BSs working cooperatively in the hotspot tier. This method will also be useful in scenarios where a handover process is initiated for a user, but the target BS set lacks the resources to serve the user coherently. Until the process is finalized, and the target BS set learns the instantaneous user channel, the user can be jointly served by the two sets of BSs, so that a disruption to service will not occur. Another use case for this detector would be a scenario where non-coherent detection may serve as a fallback or resilience mechanism-for example, during rapid channel fluctuations or sudden fading events-when coherent CSI becomes temporarily unreliable or unavailable.
- **Non-coherent primary BS with coherent assistance**: In the second scenario, we focus on a user employing differential encoding, served by a non-coherent BS that utilizes differential detection. Adding distant non-coherent BSs provides little benefit, as noted in [18], but a distant coherent BS could still support the system by working with instantaneous channels, even if the estimated channel quality is not ideal. In this study, we explore how a coherent BS can offer additional support to a non-coherent system while maintaining differential detection at the CPU. As this detector is a variation of the CNC detector, we refer to this detector as the *differential CNC detector*.

The above dual analysis examines both configurations, thereby enabling a comprehensive characterization of the performance gains achievable through coherent and non-coherent BS cooperation.

The ability to opportunistically extract gains from a mixed set of BSs—regardless whether they provide coherent or non-coherent support-enables greater adaptability and resilience in practical deployment scenarios, especially where single-mode cooperation cannot be guaranteed. The proposed detectors are especially relevant in scenarios where a user is served by BSs with varying CSI capabilities, but not enough BSs support the same cooperation mode (e.g., all coherently or all non-coherently), rendering conventional cooperative schemes ineffective. The proposed joint detection approach addresses this limitation by offering a practical solution for those users as well as the users in constrained conditions-such as those at the cell edge, with limited CSI, or in a deep fade-where performance degradation requires timely support. Moreover, hybrid detection is well-suited for beyond-5G systems where infrastructure components operate with varying levels of CSI and where there is a critical need to balance performance with resource and overhead efficiency. For instance, in cell-free massive MIMO networks [24], nearby BSs with accurate CSI can contribute coherently, while distant BSs with only statistical CSI can contribute non-coherently. Recent studies have also investigated the integration of cell-free massive MIMO with emerging paradigms such as reconfigurable intelligent surfaces (RIS) and unmanned aerial vehicle (UAV) [30,31,32,33], demonstrating the flexibility of distributed architectures in supporting heterogeneous devices and service requirements. These developments underscore the importance of hybrid detection and cooperation strategies, which motivate our proposed CNC and differential CNC detectors for scenarios with heterogeneous CSI availability. Accordingly, the joint detection framework introduced in this paper lays the foundation for adaptive, overhead-aware receiver designs that are expected to become increasingly important as wireless networks continue to evolve. In this context, RIS-assisted communications [34] and UAV-assisted systems [35] are natural application domains, where the CPU can apply the proposed detectors to combine instantaneous CSI from ground BSs with non-coherent contributions from RIS or UAV nodes that typically lack precise CSI.

The proposed detectors consider *L* contiguous symbol times and perform an exhaustive search over all possible symbol combinations to detect the transmitted symbol sequence. While the detectors, which are based on the maximum likelihood (ML) criterion, are straightforward, the mathematical analysis proves to be challenging. Hence, while an exact analysis of error performance is challenging, we provide upper bounds on the average error probability using the exact block error probability expressions. Consequently, this paper offers the following key contributions:

- We formulate two novel detectors where BSs cooperate to detect user signals having two levels of user channel information. Specifically, one group of BSs accesses instantaneous CSI, while a second group possesses only long-term channel information of the user. User signals are detected using either the CNC detector or the differential CNC detector, which are derived using the ML criterion. It is important to emphasize that the proposed detectors are not derived based on any assumptions on the channel model, and remain model-agnostic, thereby possessing the flexibility to operate across a range of fading environments depending on the system conditions. As explained later in the text, one part of the detectors operate with access to finer channel characteristics such as correlation structures or line-of-sight (LOS) component strengths, while the other does not and therefore contributes to the detection process based solely on the received channel powers. Due to this setup, the proposed detectors are not optimized for such channels; however, they offer the best performance possible with the available information and remain applicable under these conditions.
- Assuming correlated Rayleigh channels, we derive analytical expressions to determine the pairwise block error probability of a user when detected using the CNC detector and the differential CNC detector. Based on the derived pairwise error probability expression we provide an upper bound on the average block error probability of the CNC detector.
- Based on numerical results, we identify the following key insights:
  1. The CNC detector outperforms coherent detection at a single BS, with its performance gains increasing as the user moves closer to the non-coherent BS.
  2. The differential CNC detector surpasses the error performance over non-coherent differential detection at a single BS, with performance gains increasing as the user moves closer to the coherent BS.
  3. The performance degradation due to correlation at the BS antennas is minimal for both the CNC and differential CNC detectors, highlighting their robustness under such conditions.

The rest of the paper is structured as follows: Section 2 outlines the system model used in this analysis. In Section 3, we present the CNC detector, followed by Section 4, where we analyze the error performance of the proposed detector. Section 5 presents the differential CNC detector and derives an expression to calculate the pairwise block error probability. Section 6 analyzes key system-level factors. Numerical examples are given in Section 7, and the paper concludes in Section 8.

*Notation:* We use ${\left(\cdot \right)}^{*}$ for complex conjugate, ${\left(\cdot \right)}^{T}$ for transpose, and ${\left(\cdot \right)}^{H}$ for conjugate transpose. The m×n null matrix is indicated by $0_{m\times n}$ and $I_{m}$ denotes an m×m identity matrix. R is the set of real numbers. P[·] denotes probability whereas $F_{X}\left(x\right)$ denotes the cumulative distribution function (CDF) of *X* evaluated at *x*.

## 2. System Model

**Figure 1:**
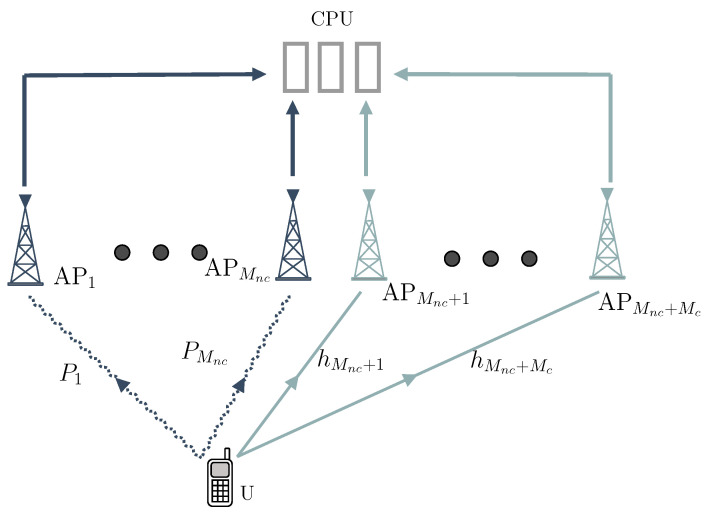
System model. The *i*th AP is denoted by ${\mathrm{AP}}_{i}$. Single antenna APs are shown to simplify the figure. ${\mathrm{AP}}_{1}$ to ${\mathrm{AP}}_{M_{nc}}$ have only the long-term channel information of the user whereas ${\mathrm{AP}}_{M_{nc}+1}$ to ${\mathrm{AP}}_{M_{nc}+M_{c}}$ possess instantaneous user channel information.

This section introduces the system model employed in the rest of the paper. We examine the uplink of a communication network where a total of $M_{nc}+M_{c}$ antennas ($M_{nc}$ non-coherent antennas and $M_{c}$ coherent antennas) collaborate to serve a user as shown in Figure 1. These antennas may be part of multiple co-located multi-antenna BSs, where at least one BS operates coherently, and the rest operate non-coherently. Alternatively, they could be distributed throughout the service area, functioning as single-antenna access points (APs). For illustrative purposes, Figure 1 employs single-antenna APs, which simplifies the problem formulation and improves notational clarity, thereby allowing a clear distinction between long-term and instantaneous user channels available at each antenna. Among these antennas, an antenna $m\in [1,\ldots M_{nc}]$ possesses only long-term information regarding the user channel whereas an antenna $m\in [M_{nc}+1,\ldots M_{nc}+M_{c}]$ obtains instantaneous CSI regarding the user channel. This setup is depicted in Figure 1, in which we have shown the possession of long-term channel information by dotted lines, and the possession of instantaneous CSI by solid lines. We define long-term channel information as the combined effect of distance-based gain and shadow fading. Consequently, the first set of antennas will aid in user symbol detection non-coherently, while the second set of antennas will aid detection coherently. We consider that the desired user is equipped with a single antenna and all $M_{nc}+M_{c}$ antennas are connected to a CPU through a delay-free fronthaul network. In this work, ideal fronthaul links with negligible latency are assumed to focus on the core design of the joint coherent and non-coherent detection framework. In practical deployments, fronthaul limitations such as transmission delays and bandwidth constraints could impact performance, particularly by affecting the availability and aging of CSI.

In this scenario, the vertically stacked received signal vectors at time instant *l*, for the non-coherent and coherent antenna groups are expressed, respectively, as

(1) $$r_{nc,l}=h_{nc}s_{l}+n_{nc,l},\;r_{nc,l},h_{nc},n_{nc,l}\in C^{M_{nc}\times 1},$$

and

(2) $$r_{c,l}=h_{c}s_{l}+n_{c,l},\;\;r_{c,l},h_{c},n_{c,l}\in C^{M_{c}\times 1}.$$

Here $s_{l}$ denotes the transmitted data symbol at time instant *l*. The corresponding noise vectors at the non-coherent and coherent antennas are $n_{nc,l}={\left[n_{1,l},\ldots ,n_{M_{nc},l}\right]}^{T}$ and $n_{c,l}={\left[n_{M_{nc}+1,l},\ldots ,n_{M_{nc}+M_{c},l}\right]}^{T}$, respectively, with $n_{m,l}$ representing the noise at antenna *m* at time instant *l*. We assume that each noise component follows a complex Gaussian distribution, characterized by a zero mean and a variance of $\sigma^{2}$. In (1) and (2), the channel vectors $h_{nc}={\left[h_{1},\ldots ,h_{M_{nc}}\right]}^{T}$ and $h_{c}={\left[h_{M_{nc}+1},\ldots ,h_{M_{nc}+M_{c}}\right]}^{T}$ collect the unknown channel coefficients from the user to the non-coherent antennas and known channel coefficients from the user to the coherent antennas, respectively. In these channel vectors, $h_{m}$ represents the channel from the user to antenna *m*.

Note that we intentionally refrain from specifying statistical properties of the channel vectors to maintain generality. The proposed detection framework is designed to be flexible and broadly applicable to many propagation environments. As the analysis progresses, we introduce specific assumptions-only when needed to support tractable derivations or provide simulation results. Importantly, the proposed detector does not rely on a fixed channel model and can operate under diverse propagation conditions, making it well-suited for deployment in heterogeneous network environments.

## 3. CNC Detector

The first main contribution of this paper, which is the design of the CNC detector is presented in this section. We use the ML estimation criterion to derive the CNC detector.

Let us first consider an observation window of *L* time steps, and define $s=[s_{1},s_{2}\ldots ,s_{L}]$ as the sequence of transmitted symbols and the matrix $R=[r_{1},r_{2},\ldots ,r_{L}]\in$$C^{(M_{nc}+M_{c})\times L}$, where $r_{l}={\left[r_{nc,l}^{T},r_{c,l}^{T}\right]}^{T}$, as the joint received signal matrix associated with this block. As the received signals at coherent and non-coherent antennas are independent, the joint probability distribution function (PDF) of the received signals at time instant *l* for coherent (i.e., $r_{c,l}$) and non-coherent (i.e., $r_{nc,l}$) antennas conditioned on the transmitted symbol sequence s, and the channel coefficients $h_{c}$ and $h_{nc}$ follows a multivariate Gaussian distribution with joint PDF given by

(3) $$\begin{array}{c} f_{R}\left(R|h_{c},h_{nc},s\right)=\frac{\exp(-\sum_{l=1}^{L}\left(\frac{{\left|\left|r_{c,l}-h_{c}s_{l}\right|\right|}^{2}}{\sigma^{2}}+\frac{{\left|\left|r_{nc,l}-h_{nc}s_{l}\right|\right|}^{2}}{\sigma^{2}}\right))}{{\left(\pi \sigma^{2}\right)}^{\left(M_{nc}+M_{c}+M_{c}\right)L}}. \end{array}$$

In (3), we assume additive white Gaussian noise with identical variance $\sigma^{2}$ at each antenna. We consider that the channel coefficients to both coherent and non-coherent antennas remain constant over the *L* time-steps considered, making a subscript of *l* unnecessary.

We define the base signal constellation denoted by M, to represent the set of symbols available for transmission at a single time instant. To model transmissions across *L* time instants jointly, we further introduce the extended constellation, $M^{L}$, defined as the set of all ordered *L*-tuples $\left(s_{1},s_{2},\ldots s_{L}\right)$ where each $s_{l}\in M$. Thus, $M^{L}$ encompasses all possible sequences of transmitted symbols over *L* consecutive uses of the channel. Now, based on (3), the ML detection problem is given as,

(4) $$s^{\mathrm{ML}}=\underset{\bar{s}\in M^{L}}{\mathrm{argmax}}\;\;f_{R}\left(R|h_{c},\bar{s}\right),$$

where $\bar{s}$ is the hypothesis for the transmitted symbols and $s^{\mathrm{ML}}$ is the detected symbol sequence according to the ML principle. In order to apply the ML principle to (3), we first need to eliminate $h_{nc}$, which is unknown at the CPU. One approach to do this is to average $f_{R}\left(R|h_{c},h_{nc},s\right)$ over the unknown channel $h_{nc}$ [11]. To accomplish this, we must have prior knowledge of the distribution of $h_{nc}$. However, non-coherent processing operates under the assumption that only channel powers are accessible at the antennas, and therefore the CPU lacks awareness regarding the channel properties. Given this lack of information, the most rational approach for decision-making is to rely on a model that solely utilizes the available knowledge, namely, the channel powers. Hence, we presume that the channels are independent and Rayleigh distributed across the antennas, i.e., $h_{nc}∼\mathrm{CN}\left(0,P_{nc}\right)$. Note that the diagonal, non-coherent long-term channel information matrix, $P_{nc}$, is defined as $P_{nc}=\mathrm{diag}\left(P_{1},\ldots ,P_{M_{nc}}\right)$ where $P_{m}$ denotes the aggregation of distance-dependent path gain and shadow fading between the user and antenna *m*. While no such assumption was made for the coherent antennas, we have assumed that the non-coherent antennas are uncorrelated. In reality, correlations may exist among the non-coherent antennas. However, since the detector can only utilize the information available at the BSs, the detector is developed based on the model outlined above.

Given these assumptions, we obtain an expression for $f_{R}\left(R|h_{c},s\right)$ as expressed in (5).

(5) $$f_{R}\left(R|h_{c},s\right)=\frac{\prod_{m=1}^{M_{nc}}\xi_{m}^{2}\sigma^{2}e^{-\frac{1}{\sigma^{2}}\sum_{l=1}^{L}\left(r_{c,l}^{H}r_{c,l}+r_{nc,l}^{H}r_{nc,l}\right)}}{{\left(\pi \sigma^{2}\right)}^{L\left(M_{c}+M_{nc}\right)}\det\left(P_{nc}\right)}\exp\left(\frac{1}{\sigma^{2}}\left(\sum_{l=1}^{L}\left(r_{c,l}^{H}h_{c}s_{l}+h_{c}^{H}r_{c,l}s_{l}^{*}-h_{c}^{H}s_{l}^{*}s_{l}h_{c}\right)+{\left|\left|\sum_{l=1}^{L}\Xi r_{nc,l}s_{l}^{*}\right|\right|}^{2}\right)\right).$$

In (5), we have introduced the diagonal weighting matrix $\Xi =\mathrm{diag}\left(\xi_{1},\ldots ,\xi_{M_{nc}}\right)$ with weighting factors defined as

(6) $$\xi_{m}={\left(\frac{P_{m}}{\sigma^{2}+P_{m}\sum_{l=1}^{L}s_{l}^{*}s_{l}}\right)}^{1/2}.$$

In order to simplify the decoding process, we assume that the power of the transmit symbols is constant in the utilized modulation scheme (as is usual in non-coherent systems [10,11,12,13]), more specifically ${∥s∥}^{2}=1\forall s\in M$. To adhere to such a criterion, we can employ either $M_{\mathrm{PSK}}$-ary phase shift keying (PSK) or $M_{\mathrm{PSK}}$-ary differential PSK modulation schemes [36]. Here and in the next section, we discuss the CNC detector using $M_{\mathrm{PSK}}$-ary modulation, and later, in Section 5 we will examine its application to $M_{\mathrm{PSK}}$-ary differential PSK. Hence, for the CNC detector the signal constellation, M, can be expressed as

(7) $$M=\left\{e^{j2\pi i/M_{\mathrm{PSK}}},i=0,...,M_{\mathrm{PSK}-1}\right\}.$$

As a result, the value of $\xi_{m}$ in (6) could be simplified as

(8) $$\xi_{m}={\left(\frac{P_{m}}{\sigma^{2}+LP_{m}}\right)}^{1/2}.$$

Note that $\xi_{m}$ in (8), which weights the non-coherent antennas relative to the long-term power at that antenna, shares a similar structure to the weighting coefficients utilized for the non-coherent antennas as in [18].

By considering the log-likelihood of (5) and disregarding terms that do not depend on the hypothesis, $\bar{s}$, we can reformulate the detection problem as

(9) sML=argmaxs¯∈MLRe∑l=1Lrc,lHhcs¯l︸Coherentcomponent+∑l=2L∑j=1l−1rnc,lHΞ2rnc,js¯ls¯j∗︸Non-coherentcomponent,

in which we have utilized the fact that ${\bar{s}}_{l}^{*}{\bar{s}}_{l}=1$, since each symbol ${\bar{s}}_{l}$ is an element of the constant-modulus transmit vector $\bar{s}$ with ${∥\bar{s}∥}^{2}=1$.

Disregarding the non-coherent component in (9) and restricting the observation window to a single time slot (L=1), simplifies the detection rule to the conventional coherent ML detection (and therein the maximum ratio combining receiver). The non-coherent component, which correlates the received signals at different time instants, closely resembles the ML detector for differential encoding outlined in [18]. Note that, due to the weighting matrix Ξ, which weights each non-coherent antenna according to (8) the signals received at the non-coherent antennas are scaled in accordance with the power received at each antenna. Consequently, in the CNC detector, the non-coherent antennas which receive a higher power from the user will exert a greater influence on the final detection outcome. This method of weighting non-coherent antennas resembles the maximum ratio combining for coherent antennas, where the signals from different antennas are weighted according to the SNR of each branch, allocating more weight to signals with a higher SNR.

The coherent and non-coherent components in (9) approach symbol detection differently.

(a) Coherent component: Evaluates which symbol at each time-step maximizes the hypothesis, thereby verifying if the symbol at each time-step is correct (${\bar{s}}_{l}=s_{l}$).
(b) Non-coherent component: Checks whether the product between each pair of symbol hypotheses at each time-step yields the correct result, i.e., ${\bar{s}}_{j}{\bar{s}}_{l}^{*}=s_{j}s_{l}^{*}$, thereby maximizing the overall hypothesis. Notably, this may still hold true even if ${\bar{s}}_{l}\neq s_{l}$ and ${\bar{s}}_{j}\neq s_{j}$.

While the coherent component tries to alleviate single time-step errors, the non-coherent component aims to correct multi-symbol errors by ensuring that the products of symbol pairs match expected outcomes across time-steps. Essentially, CNC chooses the symbol set that best meets both criteria. As such, the performance improvement of the CNC detector is largely attributed to its ability to account for both these factors, with the emphasis on each one determined by the power levels received at the respective BSs (coherent or non-coherent). If the user can deliver greater power to the non-coherent BSs, then more weight is placed on ensuring that the multiplications are correct. However, when more power is directed to the coherent BSs, the priority shifts to detecting individual symbols accurately.

Note that, when L=1, the non-coherent term disappears resulting in the coherent ML detector. As such, to implement the CNC detector, we need L≥2. In the remainder of this paper we use (9) with L≥2 and consider different user locations to analyze the performance of the CNC detector.

## 4. Error Analysis for the CNC Detector

In this section, we analyze the error performance of the CNC detector in (9).

Given that the proposed detector examines *L* consecutive time-steps to jointly determine the transmitted symbols, we evaluate the probability of erroneously detecting the symbols in the detection period. As such we refer to this probability as the *block error probability*. We first derive an analytical formula for the pairwise block error probability, where the symbol set $s=[s_{1},s_{2},\ldots ,s_{L}]$ is erroneously decoded as $\hat{s}=\left[{\hat{s}}_{1},{\hat{s}}_{2},\ldots ,{\hat{s}}_{L}\right]$, and then leverage it to determine an upper bound on the average block error probability.

In the following error analysis, we model both the coherent and non-coherent channels as correlated Rayleigh channels (if a different fading model—e.g., Rician were assumed, the analytical expressions would change accordingly), with $\Sigma_{c}$ and $\Sigma_{nc}$ being the covariance matrices of the coherent channel, $h_{c}$ and the non-coherent channel, $h_{nc}$, respectively. It is worth noting that we designed the system under the assumption that the non-coherent channels were independent, based on the fact that only long-term user power information was accessible at the non-coherent antennas, leaving correlation matrices unavailable. As a result, when correlation exists in the non-coherent channels, the detector is not fully matched to the actual channel model. However, simulation results presented later in the manuscript demonstrate that the proposed detector continues to operate effectively even in the presence of channel correlation, highlighting its robustness and adaptability in realistic settings.

### 4.1. Pairwise Block Error Probability

From (9), the pairwise block error probability of deciding on $\hat{s}$ when s was transmitted can be formulated as

(10) Ps→s^=PRe∑l=1Lrc,lHhcs^l+∑l=2L∑j=1l−1rnc,lHΞ2rnc,js^ls^j∗>Re∑l=1Lrc,lHhcsl+∑l=2L∑j=1l−1rnc,lHΞ2rnc,jslsj∗.

By employing the constants,

(11) $$\begin{array}{ccc} a_{l} & =s_{l}^{*}{\hat{s}}_{l}-1,\; & b_{l}={\hat{s}}_{l}-s_{l},\\ d_{l,j} & =s_{l}^{*}s_{j}{\hat{s}}_{l}{\hat{s}}_{j}^{*}-1,\; & e_{l,j}=s_{j}{\hat{s}}_{l}{\hat{s}}_{j}^{*}-s_{l},\\ f_{l,j} & =s_{l}^{*}{\hat{s}}_{l}{\hat{s}}_{j}^{*}-s_{j}^{*},\; & g_{l,j}={\hat{s}}_{l}{\hat{s}}_{j}^{*}-s_{l}s_{j}^{*}, \end{array}$$

we reorganize the expression in (10) to $P_{s\to \hat{s}}=P\left[T>0\right]$, where the full expression for the pairwise test statistic [37,38], *T*, is given in (12).

(12) T=Re∑l=1LalhcHhc+blnc,lHhc+∑l=2L∑j=1l−1dl,jhncHΞ2hnc+el,jnnc,lHΞ2hnc+fl,jhncHΞ2nnc,j+gl,jnnc,lHΞ2nnc,j.

Note that, in (12), we have decomposed the received signal at the coherent and non-coherent antennas using (2) and (1), respectively. This decomposition allows us to clearly separate the user channel-related terms from the noise components.

At first glance, (12) appear quite complex, as it involves the summation of multiple products containing both channel and noise terms. However, with some strategic rearrangement and considerable algebra, we transform it into a more tractable quadratic form in the following analysis. We begin by representing *T* in matrix form, which enables us to derive an expression for the CDF of *T*. Leveraging this CDF, the pairwise block error probability of deciding on $\hat{s}$ when s was transmitted can be obtained by

(13) $$P_{s\to \hat{s}}=1-F_{T}\left(0\right).$$

In order to represent *T* in matrix form, we begin by introducing the vectors

(14) $$\eta_{c}=\left[\begin{array}{c} h_{c}\\ n_{c,1}\\ \vdots \\ n_{c,L} \end{array}\right]\begin{array}{c} \in C^{\left(L+1\right)M_{c}\times 1} \end{array},\;\eta_{nc}=\left[\begin{array}{c} \Xi h_{nc}\\ \Xi n_{nc,1}\\ \vdots \\ \Xi n_{nc,L} \end{array}\right]\begin{array}{c} \in C^{\left(L+1\right)M_{nc}\times 1} \end{array},$$

and the matrices

(15) $$G_{c}=\left[\begin{array}{cccc} \sum_{l=1}^{L}\left(a_{l}+a_{l}^{*}\right)I_{M_{c}} & b_{1}^{*}I_{M_{c}} & \ldots & b_{L}^{*}I_{M_{c}}\\ b_{1}I_{M_{c}} & 0_{M_{c}} & \ldots & 0_{M_{c}}\\ \vdots & \vdots & \ddots & \vdots \\ b_{L}^{*}I_{M_{c}} & 0_{M_{c}} & \ldots & 0_{M_{c}} \end{array}\right]\begin{array}{c} \in C^{\left(L+1\right)M_{c}\times \left(L+1\right)M_{c}} \end{array},$$

and $G_{nc}\begin{array}{c} \in C^{\left(L+1\right)M_{nc}\times \left(L+1\right)M_{nc}} \end{array}$,

(16) $$\begin{array}{c} G_{nc}=\left[\begin{array}{ccccccc} \sum_{l=2}^{L}\sum_{j=1}^{l-1}\left(d_{l,j}+d_{l,j}^{*}\right)I_{M_{nc}} & \sum_{k=2}^{L}f_{k,1}I_{M_{nc}} & \left(\begin{array}{c} \sum_{k=3}^{L}f_{k,2}\\ +e_{2,1}^{*} \end{array}\right)I_{M_{nc}} & \ldots & \left(\begin{array}{c} \sum_{k=l+1}^{L}f_{k,l}\\ +\sum_{j=1}^{l-1}e_{l,j}^{*} \end{array}\right)I_{M_{nc}} & \ldots & \sum_{j=1}^{L-1}e_{L,j}^{*}I_{M_{nc}}\\ \sum_{k=2}^{L}f_{k,1}^{*}I_{M_{nc}} & 0_{M_{nc}} & g_{2,1}^{*}I_{M_{nc}} & \ldots & g_{l,1}^{*}I_{M_{nc}} & \ldots & g_{L,1}^{*}I_{M_{nc}}\\ \left(\sum_{k=3}^{L}f_{k,2}^{*}+e_{2,1}\right)I_{M_{nc}} & g_{2,1}I_{M_{nc}} & 0_{M_{nc}} & \ldots & g_{l,2}^{*}I_{M_{nc}} & \ldots & g_{L,2}^{*}I_{M_{nc}}\\ \vdots & \vdots & \vdots & \ddots & \vdots & \vdots & \vdots \\ \left(\sum_{k=l+1}^{L}f_{k,l}^{*}+\sum_{j=1}^{l-1}e_{l,j}\right)I_{M_{nc}} & g_{l,1}I_{M_{nc}} & \ldots & \ldots & 0_{M_{nc}} & \ldots & g_{L,l}^{*}I_{M_{nc}}\\ \vdots & \vdots & \vdots & \vdots & \vdots & \vdots & \vdots \\ \left(f_{L,L-1}^{*}+\sum_{j=1}^{L-2}e_{L,j}\right)I_{M_{nc}} & g_{L-1,1}I_{M_{nc}} & \vdots & \vdots & \vdots & 0_{M_{nc}} & g_{L,L-1}^{*}I_{M_{nc}}\\ \sum_{j=1}^{L-1}e_{L,j}I_{M_{nc}} & g_{L,1}I_{M_{nc}} & g_{L,2}I_{M_{nc}} & \ldots & \ldots & g_{L,L-1}I_{M_{nc}} & 0_{M_{nc}} \end{array}\right]. \end{array}$$

Now, expressing *T* in matrix format yields

(17) $$T=\eta^{H}G\eta ,$$

where $\eta ={\left[\eta_{c}^{T},\eta_{nc}^{T}\right]}^{T}$ and

(18) $$G=\left[\begin{array}{cc} G_{c} & 0_{\left(L+1\right)M_{c}\times \left(L+1\right)M_{nc}}\\ 0_{\left(L+1\right)M_{nc}\times \left(L+1\right)M_{c}} & G_{nc} \end{array}\right]\begin{array}{c} \in C^{\left(L+1\right)\left(M_{nc}+M_{c}\right)\times \left(L+1\right)\left(M_{nc}+M_{c}\right)} \end{array}.$$

Let us now define $h_{c}=\Sigma_{c}^{\frac{1}{2}}u_{h_{c}}$, $h_{nc}=\Sigma_{nc}^{\frac{1}{2}}u_{h_{nc}}$, $n_{c,l}=\sigma u_{n_{c}}$, and $n_{nc,l}=\sigma u_{n_{nc}}$ where $u_{h_{c}},u_{n_{c}}∼\mathrm{CN}\left(0,I_{M_{c}}\right)$, $u_{h_{nc}},u_{n_{nc}}∼\mathrm{CN}\left(0,I_{M_{nc}}\right)$. We next introduce the covariance matrix of η, $\Sigma_{\eta }$,

(19) $$\begin{array}{cc} \; & \Sigma_{\eta }=\\ \; & \left[\begin{array}{cccc} \Sigma_{c} & 0_{M_{c}\times M_{c}L} & 0_{M_{c}\times M_{nc}} & 0_{M_{c}\times M_{nc}L}\\ 0_{M_{c}L\times M_{c}} & \sigma^{2}I_{M_{c}L} & 0_{M_{c}L\times M_{nc}} & 0_{M_{c}L\times M_{nc}L}\\ 0_{M_{nc}\times M_{c}} & 0_{M_{nc}\times M_{c}L} & \Xi^{2}\Sigma_{nc} & 0_{M_{nc}\times M_{nc}L}\\ 0_{M_{nc}L\times M_{c}} & 0_{M_{nc}L\times M_{c}L} & 0_{M_{nc}L\times M_{nc}} & \sigma^{2}\Xi^{2}I_{M_{nc}L} \end{array}\right], \end{array}$$

and the vector $x=\left[u_{h_{c}}^{T},u_{n_{c}}^{T},\ldots ,u_{n_{c}}^{T},u_{h_{nc}}^{T},u_{n_{nc}}^{T},\ldots ,u_{n_{nc}}^{T}\right]\begin{array}{c} \in C^{1\times \left(L+1\right)\left(M_{nc}+M_{c}\right)} \end{array}$. We are now able to express (17) as

(20) $$\begin{array}{cc} T & =x^{H}\Sigma_{\eta }^{1/2}G\Sigma_{\eta }^{1/2}x=x^{H}\Omega^{H}\Lambda \Omega x={\tilde{x}}^{H}\Lambda \tilde{x}. \end{array}$$

In (20), to obtain the second equality we have utilized the fact that $\Sigma_{\eta }^{1/2}G\Sigma_{\eta }^{1/2}$ is Hermitian, allowing it to be expressed in terms of the unitary matrix Ω and the diagonal matrix Λ, which contain the eigenvalues of $\Sigma_{\eta }^{1/2}G\Sigma_{\eta }^{1/2}$. Note that when a complex Gaussian vector x is multiplied by a unitary vector, the resulting vector $\tilde{x}$ remains Gaussian in nature, following the same distribution characteristics as the original Gaussian vector. Therefore, $\tilde{x}∼\mathrm{CN}\left(0,I_{\left(M_{c}+M_{nc}\right)\left(L+1\right)}\right)$. Using (20), the characteristic function (CF) of *T* is given by

(21) $$\varphi_{T}\left(t\right)=E\left[e^{jtT}\right]=\prod_{i=1}^{n}\frac{1}{1-jt\lambda_{i}},$$

where $\gamma_{i}\in R$ are the eigenvalues of the matrix $G\Sigma_{\eta }$, and $n=\left(M_{c}+M_{nc}\right)\left(L+1\right)$. To obtain the final result in (21) we have used [39] (Lemma 2). Using the inverse Fourier transformation on (21), the CDF of *T* can be obtained as [36]

(22) $$\begin{array}{c} F_{T}\left(y\right)=\frac{1}{2\pi }\int_{-\infty }^{y}\int_{-\infty }^{\infty }\varphi_{T}\left(t\right)e^{-jtz}dtdz. \end{array}$$

The inversion approach in (22) can be simplified to a single numerical integral as in [40] (Equation (3.1)). This approach is well-known and can be conveniently computed using the gx2cdf(..., ‘method’, ‘imhof’) function from the Matlab toolbox ‘Generalized chi-square distribution’ [41], which implements the methodology from [40].

### 4.2. Upper Bound on the Average Block Error Probability

For the CNC detector using an $M_{\mathrm{PSK}}$ modulation over *L* time slots, there are $M_{\mathrm{PSK}}^{L}-1$ potential erroneous detections. By summing over all the possible erroneous detection combinations we can gain an upper bound on the average block error probability of CNC:

(23) $$P_{\mathrm{ave}}\leq P_{\mathrm{UB}}=\frac{1}{M_{\mathrm{PSK}}^{L}}\sum_{q\in S}\sum_{\hat{q}\in S\setminus \left\{q\right\}}P_{q\to \hat{q}},$$

where S is the set of all possible symbol sequences, $P_{\mathrm{ave}}$ is the average block error probability and $P_{\mathrm{UB}}$ is the union based upper bound for the CNC detector. However, given that this bound considers all conceivable erroneous scenarios, it remains relatively loose. Nevertheless, an approximation on the average block error probability of the CNC detector can be obtained by using the nearest-neighbor (NN) approximation.

To commence, let us first identify the NN errors for the CNC detector. For an arbitrary symbol, $s_{l}=e^{j2\pi i/M_{\mathrm{PSK}}}$, the closest erroneous detections would be $e^{j2\pi \left(i+1\right)/M_{\mathrm{PSK}}}$ and $e^{j2\pi \left(i-1\right)/M_{\mathrm{PSK}}}$. Note that, $s_{l}$ is the transmitted symbol at time instant, *l*, and is independent of $i\in [0,M_{\mathrm{PSK}}-1]$ which denotes the phase of the transmitted symbol. Nonetheless, to determine the NN error sequences for CNC, we should consider the erroneous symbol combinations that satisfy (a) and (b) given in Section 3.

- Considering (a), the NN errors occur when only $s_{l}$ is incorrectly detected as ${\hat{s}}_{l}=e^{j2\pi \left(i+1\right)/M_{\mathrm{PSK}}}$ (or ${\hat{s}}_{l}=e^{j2\pi \left(i-1\right)/M_{\mathrm{PSK}}}$), while all other symbols are correctly identified (i.e, ${\hat{s}}_{k}=s_{k},\;k\in \left[1,\ldots ,L\right]\setminus \left\{l\right\}$). Hence, there are 2L such NN sequences (For the special case of binary PSK, there are only *L* such NN sequences.).
- Considering (b), the symbol sequences that contain double errors which satisfy the multiplication ${\hat{s}}_{k}{\hat{s}}_{l}^{*}=s_{k}s_{l}^{*}$ but ${\hat{s}}_{l}=e^{j2\pi \left(i+1\right)/M_{\mathrm{PSK}}}$ (or ${\hat{s}}_{l}=e^{j2\pi \left(i-1\right)/M_{\mathrm{PSK}}}$) and ${\hat{s}}_{k}=e^{j2\pi \left(i+1\right)/M_{\mathrm{PSK}}}$ (or ${\hat{s}}_{k}=e^{j2\pi \left(i-1\right)/M_{\mathrm{PSK}}}$) are also NN error sequences.

By denoting the NN error sequences of a particular symbol sequence q as $N_{q}$, we can formulate an approximation on the average block error probability as

(24) $$P_{\mathrm{ave}}∼P_{\mathrm{NN}}=\frac{1}{M_{\mathrm{PSK}}^{L}}\sum_{q\in S}\sum_{\hat{q}\in N_{q}}P_{q\to \hat{q}},$$

where $P_{\mathrm{NN}}$ is the NN approximation for the CNC detector.

In Section 7, we provide simulations demonstrating that the analytical CDF obtained by inverting (21) matches perfectly with the numerically generated CDF of *T*. We also present simulations that demonstrate the effectiveness of (23) and (24).

## 5. CNC Detector with Differential Encoding

Earlier, in Section 3, we introduced the CNC detector based on $M_{\mathrm{PSK}}$-ary PSK, which can also be adapted to operate with $M_{\mathrm{PSK}}$-ary differential PSK, referred to as the differential CNC detector. In this section, we present the differential CNC detector.

### 5.1. Differential Encoding

We begin by explaining the differential encoding process, which serves as a foundation for introducing the differential CNC detector. At each time step, *l*, the user transmits a symbol, $s_{l}$, which is differentially encoded via

(25) $$s_{l}=m_{l}s_{l-1},\;s_{1}=1.$$

The information symbols, $m_{l}$, are drawn from an $M_{\mathrm{PSK}}$-ary PSK constellation, M, as defined in (7).

### 5.2. Differential CNC Detector

With differential encoding, the ML detection problem in (4) is reformulated as

(26) $$s^{\text{ML-d}}=\underset{\bar{s}\in M^{L},\;{\bar{s}}_{1}=1}{\mathrm{argmax}}\;\;f_{R}\left(R|h_{c},\bar{s}\right).$$

Note that, the key difference between (4) and (26) is that (26) assumes ${\bar{s}}_{1}=1$, while (4) does not, as there is no differential encoding involved in the former. Hence, following the same procedure outlined in Section 3, the final detection problem will be given by

(27) sML-d=argmaxs¯∈ML,s¯1=1Re∑l=2Lrc,lHhcs¯l︸Coherentcomponent+∑l=2L∑j=1l−1rnc,lHΞ2rnc,js¯ls¯j∗︸Non-coherentcomponent.

However, the resulting symbol set $s^{\mathrm{ML}-d}$ in (27) does not estimate the set of information symbols (i.e., $m_{l}$). In Section 3, the information symbols directly matched the transmitted symbols, eliminating the need for any further steps. Conversely, in this section, due to the differential encoding implemented at the user end, we are required to take an extra step to estimate the information symbols:

(28) $${\bar{m}}_{l}={\bar{s}}_{l}{\bar{s}}_{l-1}^{*},\;{\bar{s}}_{l}\in s^{\mathrm{ML}-d}.$$

As is evident from (28), the detection process compares consecutive symbols, allowing errors in one symbol to propagate to subsequent ones, thereby increasing overall detection errors. Consequently, the differential CNC detector, much like the non-coherent differential detector, is susceptible to error propagation.

### 5.3. Error Analysis of the Differential CNC Detector

The most basic and frequently adopted differential detection scheme is achieved with L=2. Therefore, this subsection analyzes the differential CNC detector error probability for the case of L=2.

For L=2, the first symbol, $s_{1}$, is always estimated in the immediately preceding cycle, while $s_{2}$ is estimated in the current cycle. However, there are two ways a correct detection of the information symbol, $m_{2}$, can occur.

- ${\hat{s}}_{1}=s_{1}$, and ${\hat{s}}_{2}=s_{2}$, therefore ${\hat{m}}_{2}=m_{2}$.
- ${\hat{s}}_{1}\neq s_{1}$, and ${\hat{s}}_{2}\neq s_{2}$, but ${\hat{m}}_{2}={\hat{s}}_{2}{\hat{s}}_{1}^{*}=m_{2}$.

Hence, the pairwise error probability of deciding on ${\hat{m}}_{2}$ when $m_{2}$ was transmitted is

(29) $$P_{m_{2}\to {\hat{m}}_{2}}=\left\{\begin{array}{c} P\left[T_{d}>0\;|\;{\hat{s}}_{1}=s_{1},{\hat{s}}_{2}\neq s_{2}\right],\\ P\left[T_{d}>0\;|\;{\hat{s}}_{1}\neq s_{1},{\hat{s}}_{2}=s_{2}\right],\\ P\left[T_{d}>0\;|\;{\hat{s}}_{1}\neq s_{1},{\hat{s}}_{2}\neq s_{2},{\hat{m}}_{2}\neq m_{2}\right], \end{array}\right.$$

in which the pairwise test statistic, $T_{d}$, is expressed in (30) where we have separated the user channel-related terms from the noise components, as previously done in Section 3. The derivation of (29) and (30) follows the same approach used for (10), but utilizing (27) for the differential CNC detector.

(30) Td=Res2∗s^2−1hcHhc+s^2−s2nc,2Hhc+s2∗s1s^2s^1∗−1hncHΞ2hnc+s2∗s^2s^1∗−s1∗hncHΞ2nnc,1+s1s^2s^1∗−s2nnc,2HΞ2hnc,+s^2s^1∗−s2s1∗nnc,2HΞ2nnc,1.

Despite being considerably simpler than *T* in (12), $T_{d}$ in (30) still comprises a summation of products that involve channel and noise terms. However, it shares a similar structure with (12), which permits us to transform it into a quadratic form as in Section 3.

By defining $\eta_{d}={\left[h_{c}^{T},n_{c,2}^{T},\Xi h_{nc}^{T},\Xi n_{nc,1}^{T},\Xi n_{nc,2}^{T}\right]}^{T}\begin{array}{c} \in C^{\left(2M_{c}+3M_{nc}\right)\times 1} \end{array}$ with the covariance matrix

(31) $$\begin{array}{c} \Sigma_{\eta_{d}}=\left[\begin{array}{cccc} \Sigma_{c} & 0_{M_{c}\times M_{c}} & 0_{M_{c}\times M_{nc}} & 0_{M_{c}\times 2M_{nc}}\\ 0_{M_{c}\times M_{c}} & \sigma^{2}I_{M_{c}} & 0_{M_{c}\times M_{nc}} & 0_{M_{c}\times 2M_{nc}}\\ 0_{M_{nc}\times M_{c}} & 0_{M_{nc}\times M_{c}} & \Xi^{2}\Sigma_{nc} & 0_{M_{nc}\times 2M_{nc}}\\ 0_{2M_{nc}\times M_{c}} & 0_{2M_{nc}\times M_{c}} & 0_{2M_{nc}\times M_{nc}} & \sigma^{2}\Xi^{2}I_{2M_{nc}} \end{array}\right]\begin{array}{c} \in C^{\left(2M_{c}+3M_{nc}\right)\times \left(2M_{c}+3M_{nc}\right)} \end{array}, \end{array}$$

and $G_{d}$ as given in (32),

(32) $$\begin{array}{c} G_{d}=\left[\begin{array}{ccccc} \left(s_{2}^{*}{\hat{s}}_{2}+s_{2}{\hat{s}}_{2}^{*}-2\right)I_{M_{c}} & {\hat{s}}_{2}^{*}-s_{2}^{*}I_{M_{c}} & 0_{M_{c}\times M_{nc}} & 0_{M_{c}\times M_{nc}} & 0_{M_{c}\times M_{nc}}\\ \left({\hat{s}}_{2}-s_{2}\right)I_{M_{c}} & 0_{M_{c}} & 0_{M_{c}\times M_{nc}} & 0_{M_{c}\times M_{nc}} & 0_{M_{c}\times M_{nc}}\\ 0_{M_{nc}\times M_{c}} & 0_{M_{nc}\times M_{c}} & \left(s_{2}^{*}s_{1}{\hat{s}}_{2}{\hat{s}}_{1}^{*}+s_{2}s_{1}^{*}{\hat{s}}_{2}^{*}{\hat{s}}_{1}-2\right)I_{M_{nc}} & \left(s_{2}^{*}{\hat{s}}_{2}{\hat{s}}_{1}^{*}-s_{1}^{*}\right)I_{M_{nc}} & \left(s_{1}^{*}{\hat{s}}_{2}^{*}{\hat{s}}_{1}-s_{2}^{*}\right)I_{M_{nc}}\\ 0_{M_{nc}\times M_{c}} & 0_{M_{nc}\times M_{c}} & \left(s_{2}{\hat{s}}_{2}^{*}{\hat{s}}_{1}-s_{1}\right)I_{M_{nc}} & 0_{M_{nc}} & \left({\hat{s}}_{2}^{*}{\hat{s}}_{1}-s_{2}^{*}s_{1}\right)I_{M_{nc}}\\ 0_{M_{nc}\times M_{c}} & 0_{M_{nc}\times M_{c}} & \left(s_{1}{\hat{s}}_{2}{\hat{s}}_{1}^{*}-s_{2}\right)I_{M_{nc}} & \left({\hat{s}}_{2}{\hat{s}}_{1}^{*}-s_{2}s_{1}^{*}\right)I_{M_{nc}} & 0_{M_{nc}} \end{array}\right], \end{array}$$

we represent $T_{d}$ in matrix form:

(33) $$\begin{array}{cc} T_{d} & =\eta_{d}^{H}G_{d}\eta_{d}. \end{array}$$

Following the same approach as in Section 3, the CDF of $T_{d}$ can be evaluated by inverting the CF of $T_{d}$:

(34) $$\varphi_{T_{d}}\left(t\right)=\prod_{i=1}^{n_{d}}\frac{1}{1-jt\gamma_{i}},$$

where $\gamma_{i}\in R$ are the eigenvalues of the matrix $G_{d}\Sigma_{\eta_{d}}$, and $n_{d}=2M_{c}+3M_{nc}$. Similar to Section 5.3, the CDF of $T_{d}$ can be calculated using the gx2cdf(..., ‘method’, ‘imhof’) function from the Matlab toolbox ‘Generalized chi-square distribution’ [41]. In Section 7, we present simulations showing that the analytical CDF obtained using (33) aligns perfectly with the numerically computed CDF of $T_{d}$.

Note that, there is an interdependency between the symbols transmitted in the two time-steps, $s_{1}$ and $s_{2}$, as well as between the information symbol $m_{2}$ and the symbols $s_{1}$ and $s_{2}$. Moreover, as noted previously in Section 5.2, due to the inherent iterative approach, differential CNC is prone to error propagation. Systems characterized by error propagation present considerable challenges for effective error analysis [42]. As such, it is not possible to derive a generic upper bound on the average error probability for the differential CNC using the union bounding technique (or the NN approach) on the pairwise error probability, $P_{m_{2}\to {\hat{m}}_{2}}$, as was done in Section 5.3. Nonetheless, by assuming previously detected symbols are error-free (Note that this assumption is a valid approximation in the high SNR regime, where the probability of the previous symbol being detected incorrectly is low [42,43].), we are able to derive an upper bound on the average symbol error probability (SEP) by employing the union bounding technique:

(35) $$\begin{array}{c} P_{\mathrm{ave}}^{d}\leq \frac{1}{M_{\mathrm{PSK}}}\sum_{s_{2}\in M}\sum_{{\hat{s}}_{2}\in M\setminus \left\{s_{2}\right\}}P\left[T_{d}>0\;|\;{\hat{s}}_{1}=s_{1},{\hat{s}}_{2}\neq s_{2}\right]=P_{\mathrm{UB}}^{d}, \end{array}$$

where $P_{\mathrm{ave}}^{d}$ is the average SEP for the differential CNC detector and $P_{\mathrm{UB}}^{d}$ is the union based upper bound for the differential CNC detector.

In Section 7 we evaluate and compare the differential CNC detector with the CNC detector. We also provide simulations showing that (35) effectively serves as an upper bound on the average SEP for the differential CNC detector in the high SNR regime.

## 6. System-Level Considerations

In this section, we examine key system-level factors that impact the practical deployment of the proposed detection framework. Specifically, we analyze three important aspects: the computational complexity associated with the detection process, the network overhead arising from the exchange of channel information between BSs and the CPU and the performance implications of adding additional cooperative BSs—both coherent and non-coherent—to the proposed detectors. We provide comparisons for the complexity and network overhead incurred by the proposed approaches against two appropriate baseline schemes: fully coherent ML detection and fully non-coherent ML detection. These benchmarks represent the two extremes in terms of CSI availability and cooperation models, and provide a meaningful context for evaluating the proposed middle-ground joint detection approach.

To quantify the associated complexity, we decompose the computational cost into two components: (i) the number of complex operations required for channel estimation, and (ii) the complexity of ML detection. We assume that all cooperating BSs use the same channel estimation method, and that estimating the channel of the target user at any BS requires C complex operations. Consequently, the total channel estimation complexity depends on both the number of participating BSs and the type of CSI each BS provides during detection. We further characterize the ML detection complexity by the number of complex multiplications (Due to their low hardware cost, additions and subtractions are not considered [44].) required to evaluate the detection rule. Similarly, we quantify network overhead by the number of instantaneous channel coefficients that must be transmitted to the CPU in a coherence block. A detailed comparison of the three considered schemes is presented in Table 1. To emphasize that the number of cooperative antennas in fully coherent and fully non-coherent modes are different to that used in CNC and differential CNC, we denote the number of antennas in fully coherent and fully non-coherent mode as $M_{c}^{F}$ and $M_{nc}^{F}$, respectively.

The proposed scheme relaxes the need for acquiring instantaneous CSI from all cooperating BSs, resulting in a significant reduction in both signaling overhead and channel estimation complexity compared to fully coherent cooperative systems that require real-time CSI at every BS. However, this comes at the cost of a higher overhead relative to fully non-coherent cooperative systems. In Section 7, we demonstrate that, given the same overhead for channel estimation and network signaling, the proposed approach outperforms both fully coherent and fully non-coherent schemes.

It is worth emphasizing that while this work focuses on full ML detection to establish a performance benchmark for joint coherent and non-coherent processing, the exhaustive search involved can become computationally demanding with higher-order modulations or longer detection windows. To address this, low-complexity alternatives such as sphere decoding [45,46], lattice reduction-aided detection [47], or sequential search techniques [48,49] can be applied to reduce computational burden while preserving near-ML performance. Incorporating these complexity-reduction strategies into the hybrid detection framework is a promising direction for future work and practical system design.

Next, we discuss the impact of adding additional cooperative BSs-both coherent and non-coherent-on system level performance of the CNC and differential CNC detectors. From (9) and (27), it can be observed that the influence of each non-coherent antenna on the final decision metric is weighted by the power it captures. Therefore, increasing the number of non-coherent BSs does not always result in performance improvement (although performance does not degrade)-especially when the received signal powers at those BSs are uneven. In cases where one BS dominates in received power, the contribution from additional, weaker BSs becomes marginal, offering limited combining gain in the overall detection process. This phenomenon mirrors that of [18], where it was noted that the effectiveness of cooperation hinges largely on cooperating non-coherent BSs receiving nearly equal powers from the user. In contrast, adding more coherent BSs tends to yield more consistent and substantial performance gains. This is due to the phase-aligned nature of coherent combining, which allows effective signal addition even when received powers vary moderately. However, this improvement comes at the cost of higher system-level complexity: each coherent BS must perform accurate instantaneous channel estimation and transmit this information to the central processor. As a result, coherent cooperation introduces significant overhead in terms of CSI acquisition and coordination. This trade-off underscores a key strength of the joint detection scheme. The proposed detectors enable the system to opportunistically adapt to available resources, utilizing coherent BSs where CSI is reliable and supplementing with non-coherent BSs in a low-overhead, power-aware manner.

While this work focuses on the design and performance evaluation of the joint detectors, we acknowledge that a practical deployment would benefit from a more systematic strategy to classify BSs as coherent or non-coherent. Such a strategy could be based on real-time factors such as signal quality, mobility patterns, or service-level requirements. We consider this another valuable direction for future work, aimed at enhancing the adaptability and efficiency of joint cooperative detection in dynamic networks.

## 7. Numerical Results

**Figure 2:**
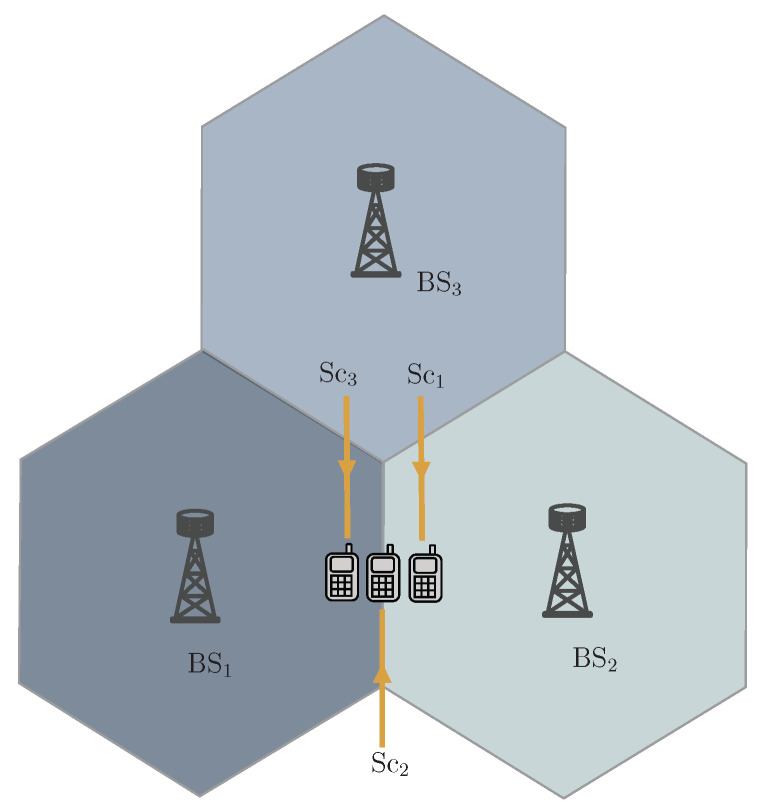
Simulation model: three hexagonal cells each equipped with a BS located at the cell center.

In this section, we evaluate the performance of the proposed CNC and differential CNC detectors through Monte Carlo simulations. The complete simulation setup and parameters are summarized in Table 2. To investigate the impact of user location on detector performance, we consider three representative user positions, also listed in Table 2. The layout of the BSs and the three user scenarios is illustrated in Figure 2. Note that since shadowing effects are not included in the channel model, the user’s position directly determines the received power levels at the BS antennas. Unless specified otherwise, the subsequent figures present the average SEP versus the received signal-to-noise ratio (SNR), comparing the performance of the proposed detectors with that of the baseline counterparts. The cooperation gain achieved by the proposed detectors across various configurations is evident from the improvements in average SEP depicted in the in those figures.

**Figure 3:**
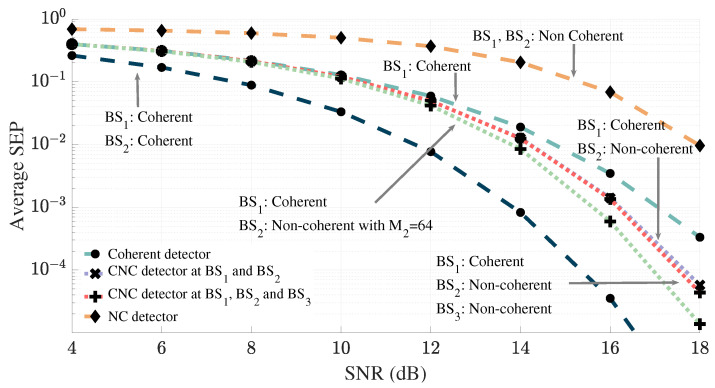
Error performance of the user: Performance comparison as the number of coherent and non-coherent antennas change.

In Figure 3, we illustrate the performance gains experienced by the user (located corresponding to ${\mathrm{Sc}}_{2}$) when signals are detected using the CNC detector, compared to detection by a non-cooperative coherent BS. Here, we assume that ${\mathrm{BS}}_{1}$ possesses instantaneous CSI of the user, while ${\mathrm{BS}}_{2}$ and ${\mathrm{BS}}_{3}$ only have access to long-term channel information. The light blue dashed curve represents the error performance of the user when detected coherently at ${\mathrm{BS}}_{1}$. If ${\mathrm{BS}}_{2}$ also had instantaneous CSI and both ${\mathrm{BS}}_{1}$ and ${\mathrm{BS}}_{2}$ were used for coherent detection, the user would obtain the error performance depicted in the dark blue curve. However, since ${\mathrm{BS}}_{2}$ only possesses long-term channel information, this level of performance is unachievable. Thus, employing the CNC detector with ${\mathrm{BS}}_{1}$ operating coherently and ${\mathrm{BS}}_{2}$ operating non-coherently results in the error performance illustrated by the light purple curve, which demonstrates a notable improvement over coherent detection alone (e.g., at SNR = 18 dB the average SEP decreases from $3.3\times 10^{-4}$ with coherent ML detection to $5.8\times 10^{-5}$ with the proposed CNC detector, corresponding to an improvement of approximately 83%). Notably, the CNC detector outperforms coherent detection while maintaining the same channel estimation and signaling overhead. The light orange dashed line represents the average SEP when both ${\mathrm{BS}}_{1}$ and ${\mathrm{BS}}_{2}$ operate in a fully non-coherent mode, relying solely on long-term channel information to cooperatively detect the user’s signal. As observed, even with cooperation, the performance of the non-coherent detector remains significantly limited.

**Figure 8:**
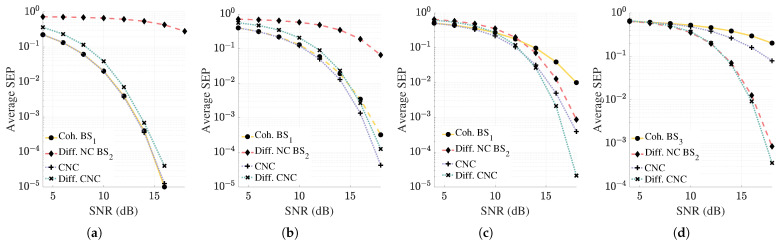
Performance comparison of CNC and differential CNC (Diff. CNC): (**a**) ${\mathrm{BS}}_{1}-{\mathrm{BS}}_{2}$ cooperation for ${\mathrm{Sc}}_{3}$. (**b**) ${\mathrm{BS}}_{1}-{\mathrm{BS}}_{2}$ cooperation for ${\mathrm{Sc}}_{2}$. (**c**) ${\mathrm{BS}}_{1}-{\mathrm{BS}}_{2}$ cooperation for ${\mathrm{Sc}}_{1}$. (**d**) ${\mathrm{BS}}_{2}-{\mathrm{BS}}_{3}$ cooperation for ${\mathrm{Sc}}_{1}$.

Figure 3 also demonstrates the error performance of the CNC detector when more than one non-coherent BS cooperates with a coherent BS to detect user signals. The orange dotted line shows the error performance when ${\mathrm{BS}}_{3}$ is also included in the CNC detector as a non-coherent BS, contributing only a very minor increase in performance compared to the CNC detector with just ${\mathrm{BS}}_{1}$ and ${\mathrm{BS}}_{2}$. This performance improvement is very small with 32 antennas at each BS, and both curves seem to be on top of each other. For lower number of antennas at each BS this improvement is more discernible. As explained in Section 6, the marginal improvement associated with incorporating ${\mathrm{BS}}_{3}$ into the detection process stems from the fact that the signals at this BS are weighted based on the received power. Due to the distant location of ${\mathrm{BS}}_{3}$ (compared to that of ${\mathrm{BS}}_{2}$), the aid supplied by ${\mathrm{BS}}_{3}$ for the CNC detector is minimal. Alternatively, doubling the antennas at ${\mathrm{BS}}_{2}$ (or adding another non-coherent BS obtaining similar power to ${\mathrm{BS}}_{2}$), rather than incorporating ${\mathrm{BS}}_{3}$, would result in a greater performance improvement as depicted by the green dotted curve. This is attributed to the higher weighting factor at ${\mathrm{BS}}_{2}$. As such, in the subsequent simulations (except Figure 8) we consider only ${\mathrm{BS}}_{1}$ and ${\mathrm{BS}}_{2}$.

**Figure 4:**
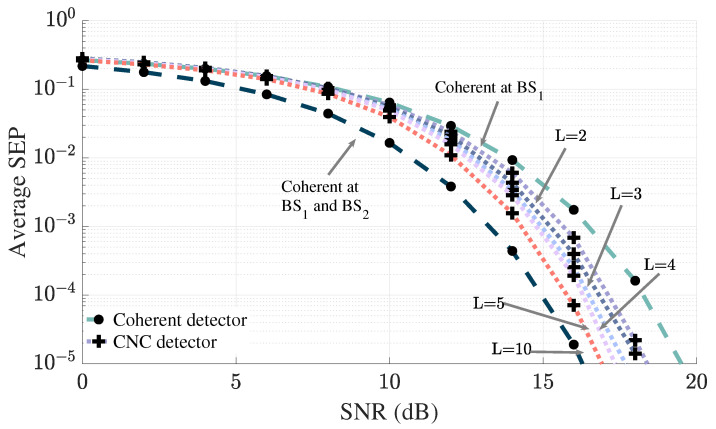
Performance of the CNC detector as *L* increases.

The error performance of the CNC detector as *L* increases is depicted in Figure 4. As expected, the trends illustrated in Figure 4 suggest that the performance gains of the CNC detector diminish with each additional increase in *L*. Additionally, it appears that as *L* tends towards infinity, the performance of the CNC detector is constrained by the error performance achievable through coherent detection at both BSs.

**Figure 5:**
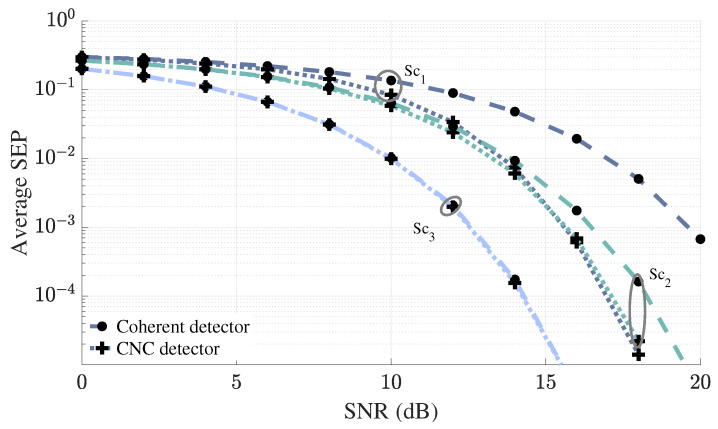
Performance gain of the CNC detector as user moves away from the non-coherent BS.

Figure 5 illustrates how the performance gains of the CNC detector vary as the user moves away from the non-coherent BS (${\mathrm{BS}}_{2}$). We observe that the performance gain of the CNC detector is highest in ${\mathrm{Sc}}_{1}$ i.e., when the user is positioned closer to ${\mathrm{BS}}_{2}$. The reason for this improvement is that being closer to ${\mathrm{BS}}_{2}$ allows it to capture stronger signals from the user, which increases the scaling factor and consequently, enhances the benefits derived from adding the non-coherent BS in the detection process. Additionally, both CNC and coherent detectors show improved performance as the user approaches the coherent BS. This improvement is due to the improved channel conditions at the coherent BS, which enables more accurate decoding of the user’s signals.

**Figure 6:**
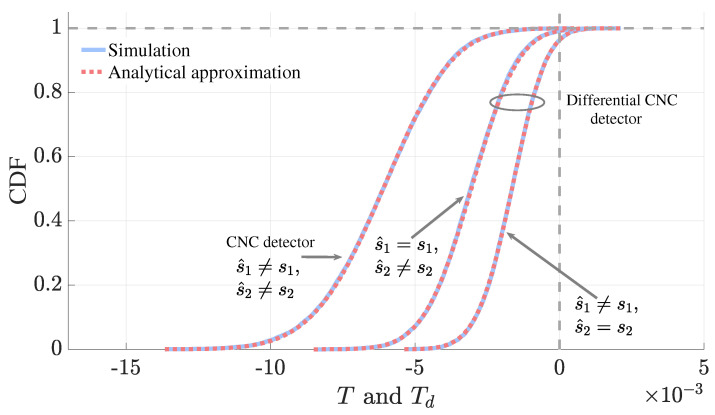
Analytical and simulated CDF of the pairwise test statistic.

In Figure 6, we demonstrate that the accuracy of the analytical expressions for the CDF of the pairwise test statistics, *T* and $T_{d}$, which were obtained via inversion of (21) and (34), respectively. For the CNC detector, we plot the CDF where both signals are detected incorrectly (i.e., ${\hat{s}}_{1}\neq s_{1}$ and ${\hat{s}}_{2}\neq s_{2}$). For the differential CNC detector, we analyze two specific error cases: first, when $s_{1}$ is correctly detected while $s_{2}$ is in error (i.e., ${\hat{s}}_{1}=s_{1},{\hat{s}}_{2}\neq s_{2}$), and second, when $s_{2}$ is correctly detected while $s_{1}$ is in error (i.e., ${\hat{s}}_{1}\neq s_{1},{\hat{s}}_{2}=s_{2}$). We observe that the derived expression aligns with the simulated CDFs in all three cases.

**Figure 7:**
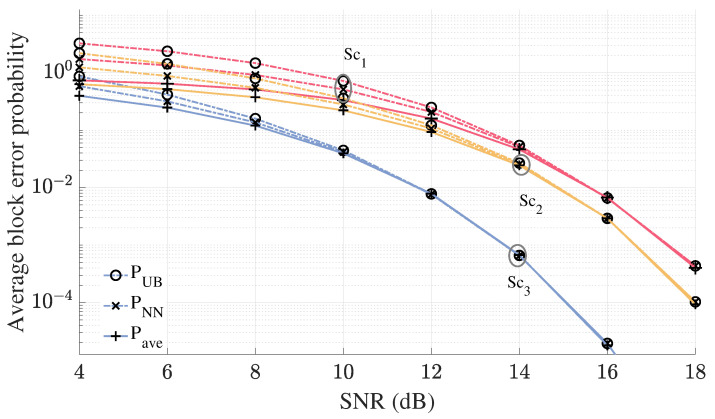
CNC detector: A comparison of simulations and bounds.

In Figure 7, the effectiveness of the union-based upper bound (23) and the NN approximation (24) on average block error probability of the CNC detector is demonstrated for the three considered scenarios. Both the upper bound and the NN approximation accurately capture the error behavior of the CNC detector for all three scenarios. Additionally, as SNR increases, both the bound and the approximation become tighter, eventually converging, with the NN approximation and union-based bound yielding the same results.

Figure 8 illustrates how CNC compares to differential CNC as the user location changes. We assume that ${\mathrm{BS}}_{1}$ and ${\mathrm{BS}}_{3}$ have access to instantaneous CSI, while ${\mathrm{BS}}_{2}$ has only long-term channel information of the user. For comparison, the error performance for single BS coherent detection (denoted by Coh. in the plots–${\mathrm{BS}}_{1}$ in Figure 8a–c, and ${\mathrm{BS}}_{3}$ in Figure 8d) and non-coherent differential detection (denoted by Diff. NC in the plots-${\mathrm{BS}}_{2}$) are also shown. Similar to Figure 5, Figure 8a–c, demonstrate the increasing performance gain of the CNC detector as the user approaches the non-coherent BS. As we progress from Figure 8a–d, the differential non-coherent detector (as well as the differential CNC detector) begins to outperform the coherent detector as the user moves farther from the coherent BS. This phenomenon likely occurs because, as the user moves away from the coherent BS, the precise channel estimates no longer yield superior error performance compared to processing the user at a nearby BS with long-term information. To achieve good performance from the non-coherent BS, differential encoding and detection are required to cancel out the channel effect. Furthermore, as the user continues to move away, the differential CNC detector begins to outperform the CNC detector. This trend is more noticeable in Figure 8d, where the distant coherent BS (${\mathrm{BS}}_{3}$) performs poorly, and using CNC with ${\mathrm{BS}}_{2}$ and ${\mathrm{BS}}_{3}$ instead of differential non-coherent detection at ${\mathrm{BS}}_{2}$ degrades the system’s error performance. However, differential CNC with ${\mathrm{BS}}_{2}$ and ${\mathrm{BS}}_{3}$ can only marginally improve performance as ${\mathrm{BS}}_{3}$ is too distant to provide significant assistance.

**Figure 9:**
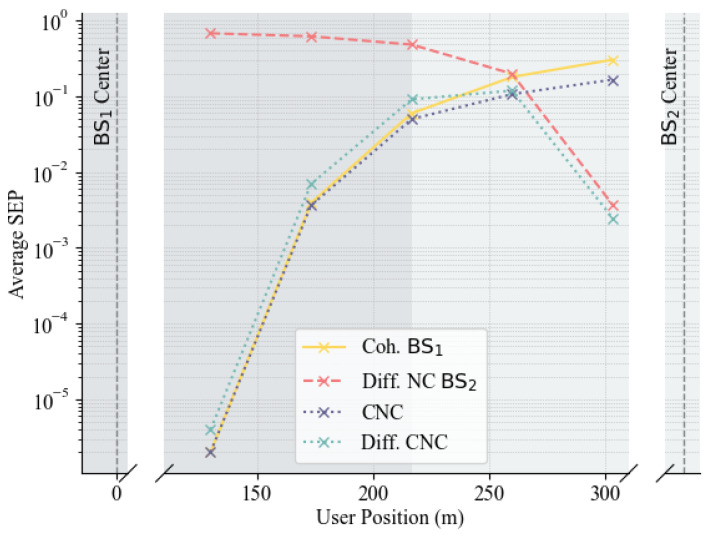
Performance of the detectors as the user moves away from the coherent BS (${\mathrm{BS}}_{1}$) towards the non-coherent BS (${\mathrm{BS}}_{2}$).

Figure 9 further demonstrates the above discussed trend in detector performance as the user moves from the vicinity of the coherent BS (${\mathrm{BS}}_{1}$) to the non-coherent BS (${\mathrm{BS}}_{2}$). The x-axis represents the user’s position along the line between the two BSs. As expected, the performance of the fully coherent detector (Coh. ${\mathrm{BS}}_{1}$) degrades as the user moves away from ${\mathrm{BS}}_{1}$ due to weaker signal strength. Conversely, the performance of the differential non-coherent detector associated with ${\mathrm{BS}}_{2}$ (Diff. NC ${\mathrm{BS}}_{2}$) improves, as it approaches ${\mathrm{BS}}_{2}$. The proposed joint detectors in particular differential CNC detector exhibit strong adaptability. These detectors effectively combine the strengths of both coherent and non-coherent processing, resulting in consistently lower average SEP compared to using either BS in isolation.

**Figure 10:**
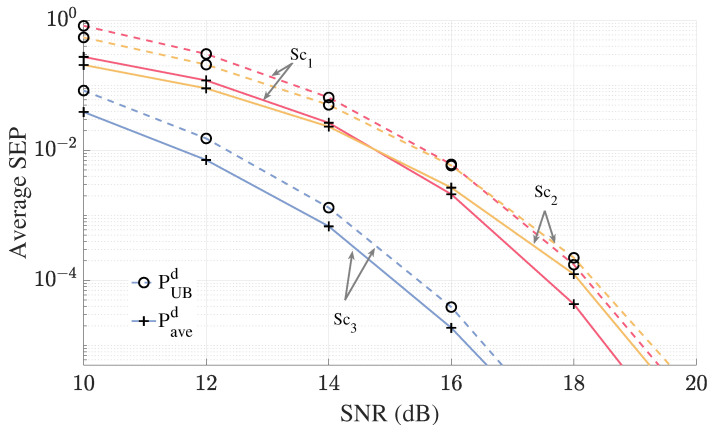
Differential CNC detector: A comparison of simulations and bounds.

Similar to Figure 7, Figure 10 highlights the effectiveness of the upper bound given in (35) in capturing the behavior of the average SEP of the differential CNC detector in the high SNR regime. The results show that the upper bound effectively captures the error behavior in all three considered cases, making it a practical tool for understanding system dynamics with differential CNC detector in high-SNR conditions.

**Figure 11:**
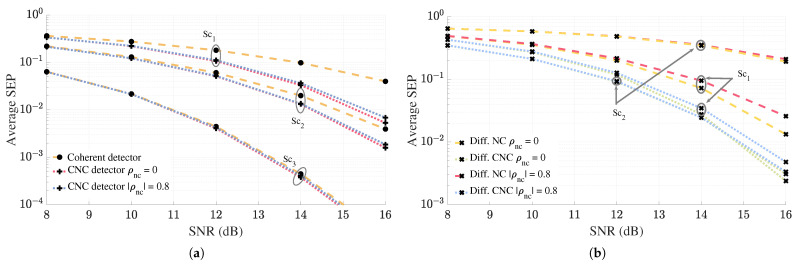
Effect of correlated channels on the proposed detectors: (**a**) CNC detector. (**b**) Differential CNC detector.

Figure 11 assesses the impact of correlation at BS antennas on the error performance of the proposed detectors. We model the user channels at both coherent and non-coherent BSs as exponentially correlated Rayleigh channels using [50]. Hence, the correlation matrix at the coherent BS is defined by ${\left(R_{c}\right)}_{i,j}=\rho_{c}^{\left|i-j\right|}$, and at the non-coherent BS we have ${\left(R_{nc}\right)}_{i,j}=\rho_{nc}^{\left|i-j\right|}$, where $\rho_{c}$ and $\rho_{nc}$ represent the correlation coefficient between neighboring receive branches at the coherent and non-coherent BS, respectively. In Figure 11a, the light orange curves show the performance of the coherent detector at ${\mathrm{BS}}_{1}$, while the pink curves depict the performance of the CNC detector if the user channels at the non-coherent BS, ${\mathrm{BS}}_{2}$, were independent. In Figure 11b, the light orange curve represents the performance of the non-coherent differential detector, while the green curve shows the performance of the differential CNC detector, both assuming independent user channels at the non-coherent BS. Since both CNC and differential CNC detectors are designed under the assumption of uncorrelated non-coherent antennas, the presence of correlation at these antennas is likely to result in some performance loss. Accordingly, when the user channels at the non-coherent BS are correlated with $\rho_{nc}=0.8\exp\left(j\pi /4\right)$, a performance drop is observed, as depicted by the blue lines (for both CNC and differential CNC) in the plots. Since the non-coherent differential detector encounters the same issue as the CNC and differential CNC detectors, it also experiences a performance loss, illustrated by the pink curve in Figure 11b. Nevertheless, we observe that, even with a mid-to-high correlation coefficient, the performance loss is minimal across all three scenarios for the CNC detector, with degradation becoming even less noticeable as the user approaches the coherent BS. For the differential CNC detector in Figure 11b we have only shown the performance curves for ${\mathrm{Sc}}_{1}$ and ${\mathrm{Sc}}_{2}$ as adding in ${\mathrm{Sc}}_{3}$ would add clutter to the plot. We observe that, for the differential CNC detector in both scenarios, the performance degradation is minimal. Similar to the CNC detector, the performance loss due to correlation between non-coherent antennas diminishes as the user moves farther from the non-coherent BS.

**Figure 12:**
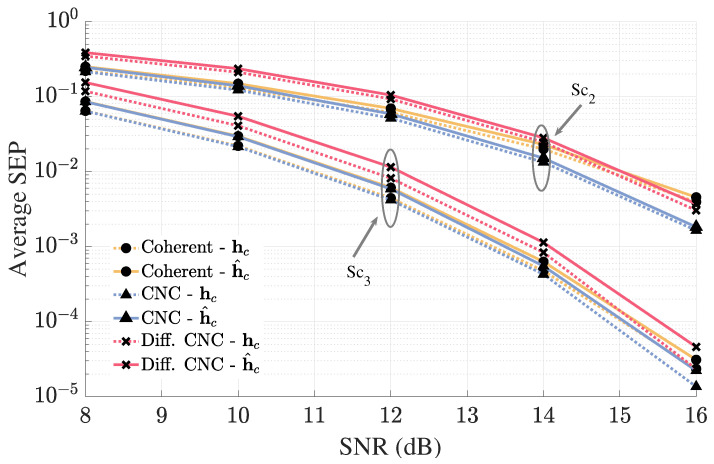
Impact of channel estimation errors on the proposed detectors. The dotted curves represent detector performance under perfect channel knowledge ($h_{c}$), while the solid curves correspond to performance when estimated channels (${\hat{h}}_{c}$) are used in the detection process.

Figure 12 illustrates the sensitivity of the proposed detectors to channel estimation errors. We model the estimation error using the framework described in [51], which captures the statistical properties of errors arising from the use of a linear minimum mean squared error (LMMSE) channel estimator. For this evaluation, the training SNR is set equal to the detection SNR to highlight the impact of estimation inaccuracies-despite this conservative assumption, the performance degradation remains low in both considered scenarios. It is important to note that, in practical systems, the training SNR is typically higher than that of the detection SNR, which would result in even smaller estimation-induced degradation. To maintain the presentation clarity, the performance curves for ${\mathrm{Sc}}_{1}$ have been omitted, and only ${\mathrm{Sc}}_{2}$ and ${\mathrm{Sc}}_{3}$ are shown. In the configuration represented by scenario ${\mathrm{Sc}}_{3}$, where coherent BSs contribute more significantly to the detection process, the system exhibits slightly increased sensitivity to estimation error, consistent with its heavier reliance on accurate coherent CSI. The behavior of ${\mathrm{Sc}}_{1}$ follows trivially, exhibiting even lower—effectively negligible—performance degradation due to the minimal influence of coherent channels in that configuration. As such, the proposed CNC and differential CNC detectors exhibit strong robustness to imperfect CSI, with performance loss closely matching that of the conventional coherent detector. It is important to emphasize that the performance degradation experienced by the proposed detectors under channel estimation errors is highly dependent on the choice of estimation algorithm. With a robust approach such as LMMSE, the degradation remains limited and aligns closely with that of the conventional coherent detector.

## 8. Conclusions

We introduced a novel detector termed the CNC detector which enables cooperation among BSs that have access to different levels of user channel information. In this setup, some BSs possess instantaneous user channel information and are able to detect user signals coherently, while others relying on long-term information of the user’s channel can detect user signals non-coherently. The CNC and differential CNC detectors combine the coherent and non-coherent methods of signal detection and enables cooperation among these two sets of BSs. We derived analytical expressions for the pairwise block error probability for both detectors and provided a tight upper bound on the average block error probability for the CNC detector. Our numerical evaluations demonstrated that the CNC detector and the differential CNC detector outperform coherent detection and non-coherent differential detection, respectively, at a single BS. Notably, the performance of the CNC detector improves as the user moves closer to BSs cooperating with long-term channel information, while the differential CNC detector shows higher gains as the user approaches BSs utilizing instantaneous CSI. Furthermore, we note that both detectors exhibited strong resilience to mid-to-high correlation at the BS antennas, and channel estimation errors highlighting their robustness in practical deployment scenarios.

## Figures and Tables

**Table 1:** Comparison of computational complexity and network overhead between the proposed detection schemes and baseline approaches.

	Channel Estimation	ML Detection	Network Overhead
Fully coherent	$CM_{c}^{F}$	$LM_{c}^{F}M_{\mathrm{PSK}}$	$M_{c}^{F}$
Fully non-coherent	-	$\frac{L(L-1)}{2}M_{nc}^{F}M_{\mathrm{PSK}}^{2}$	-
CNC	$CM_{c}$	$LM_{c}M_{\mathrm{PSK}}+\frac{L(L-1)}{2}M_{nc}M_{\mathrm{PSK}}^{2}$	$M_{c}$
Differential CNC	$CM_{c}$	$M_{c}M_{\mathrm{PSK}}+M_{nc}M_{\mathrm{PSK}}^{2}$	$M_{c}$

**Table 2:** Simulation Setup and Parameters.

Category	Details
System model	Hexagonal cell with three BSs (${\mathrm{BS}}_{1}$, ${\mathrm{BS}}_{2}$, ${\mathrm{BS}}_{3}$) at fixed positions and are equipped with $M_{1}$, $M_{2}$ and $M_{3}$ antennas, respectively.
Antennas per BS	$M_{1}=M_{2}=M_{3}=32$ *
User	Single-antenna user
User positions	Scenario 1 (${\mathrm{Sc}}_{1}$): User 20% closer to ${\mathrm{BS}}_{2}$ compared to ${\mathrm{Sc}}_{2}$ Scenario 2 (${\mathrm{Sc}}_{2}$): User equidistant from ${\mathrm{BS}}_{1}$ and ${\mathrm{BS}}_{2}$ Scenario 3 (${\mathrm{Sc}}_{3}$): User 20% farther from ${\mathrm{BS}}_{2}$ compared to ${\mathrm{Sc}}_{2}$
Modulation	QPSK (M=4)
Channel model	Independent Rayleigh fading *
Path loss	Distance-dependent path decay with path loss exponent = 4. To ensure simplicity in numerical results, only distance-based path loss was considered in the simulations. Incorporating shadow fading would have introduced variability that could obscure key performance trends. Nonetheless, as shadow fading is incorporated in the detector design, the derived detectors could be readily used in systems where shadow fading is present.
Block length	L=2 *
Detectors compared	Proposed: CNC, Differential CNC Baselines: Fully coherent ML, Fully non-coherent ML

* Unless otherwise stated.

## Data Availability

Data contained within the article.

## Abbreviations

The following abbreviations are used in this manuscript:

MIMO	Multiple-input Multiple-output
BS	Base Station
CSI	Channel State Information
CNC	Coherent/Non-coherent
ML	Maximum Likelihood
SINR	Signal-to-Interference-and-Noise Ratio
SNR	Signal-to-Noise Ratio
CPU	Unmanned Aerial Vehicle
RIS	Reconfigurable Intelligent Surfaces
AP	Access Point
PDF	Probability Distribution Function
PSK	Phase Shift Keying
CDF	Cumulative Distribution Function
CF	Characteristic Function
NN	Nearest Neighbor
SEP	Symbol Error Probability

## References

[1] Liu L., Yu W. (2018). Massive connectivity with massive MIMO-Part I: Device activity detection and channel estimation. IEEE Trans. Signal Process..

[2] Marzetta T.L. (2016). Fundamentals of Massive MIMO.

[3] Senanayake R., Yeoh P.L., Evans J.S. (2015). Performance analysis of centralized and partially decentralized co-operative networks. IEEE Trans. Commun..

[4] O’Hurley S., Tran L.N. A Comparison of the Uplink Performance of Cell-Free Massive MIMO using Three Linear Combining Schemes: Full-Pilot Zero Forcing with Access Point Selection, Matched-Filter and Local-Minimum-Mean-Square Error. Proceedings of the 2020 31st Irish Signals and Systems Conference (ISSC).

[5] Chen S., Zhang J., Zhang J., Björnson E., Ai B. (2022). A survey on user-centric cell-free massive MIMO systems. Digit. Commun. Netw..

[6] Attarifar M., Abbasfar A., Lozano A. (2020). Subset MMSE receivers for cell-free networks. IEEE Trans. Wireless Commun..

[7] Björnson E., Sanguinetti L. (2020). Scalable cell-free massive MIMO systems. IEEE Trans. Commun..

[8] Gunasekara S., Senanayake R., Smith P., Kuijper M. A Novel Partial Joint Processing Architecture for distributed Massive MIMO. Proceedings of the 95th IEEE Vehicular Technology Conference, VTC Spring 2022.

[9] Gunasekara S., Senanayake R., Smith P., Kuijper M. (2024). Zeroing Unknown Terms: A Novel Clustering Architecture for Low Network Overhead in Distributed Massive MIMO. IEEE Open J. Commun. Soc..

[10] Armada A.G., Hanzo L. A non-coherent multi-user large scale SIMO system relaying on M-ary DPSK. Proceedings of the 2015 IEEE International Conference on Communications, ICC 2015.

[11] Schenk A., Fischer R.F. Noncoherent detection in massive MIMO systems. Proceedings of the 17th International ITG Workshop on Smart Antennas, WSA 2013.

[12] Baeza V.M., Armada A.G. (2019). Non-coherent massive SIMO system based on M-DPSK for Rician channels. IEEE Trans. Veh. Technol..

[13] Morales M.J.L., Chen-Hu K., Armada A.G. Effect of Spatial Correlation on the Performance of Non-coherent Massive MIMO based on DMPSK. Proceedings of the IEEE Global Communications Conference, GLOBECOM 2021.

[14] Xie H., Xu W., Ngo H.Q., Li B. (2020). Non-coherent massive MIMO systems: A constellation design approach. IEEE Trans. Wireless Commun..

[15] Manolakos A., Chowdhury M., Goldsmith A. (2016). Energy-Based Modulation for Noncoherent Massive SIMO Systems. IEEE Trans. Wireless Commun..

[16] Manolakos A., Chowdhury M., Goldsmith A.J. CSI is not needed for optimal scaling in multiuser massive SIMO systems. Proceedings of the 2014 IEEE International Symposium on Information Theory.

[17] Chowdhury M., Manolakos A., Goldsmith A. (2016). Scaling laws for noncoherent energy-based communications in the SIMO MAC. IEEE Trans. Inf. Theory.

[18] Gunasekara S., Smith P., Kuijper M., Senanayake R. Non-coherent detection with differential modulation for distributed massive MIMO Systems. Proceedings of the 97th IEEE Vehicular Technology Conference, VTC Spring 2023.

[19] Gunasekara S., Smith P., Kuijper M., Senanayake R. Invited Paper: SNR Analysis of Differential Modulation for Distributed Massive MIMO Systems. Proceedings of the 17th IEEE International Conference on Industrial and Information Systems, ICIIS 2023.

[20] Wang L., Hanzo L. (2011). Dispensing with channel estimation: Differentially modulated cooperative wireless communications. IEEE Commun. Surv. Tutor..

[21] Xu W., Alshamary H.A., Al-Naffouri T., Zaib A. (2019). Optimal joint channel estimation and data detection for massive SIMO wireless systems: A polynomial complexity solution. IEEE Trans. Inf. Theory.

[22] Song H., Goldstein T., You X., Zhang C., Tirkkonen O., Studer C. (2022). Joint channel estimation and data detection in cell-free massive MU-MIMO systems. IEEE Trans. Commun..

[23] Karataev A., Forsch C., Cottatellucci L. (2024). Bilinear Expectation Propagation for Distributed Semi-Blind Joint Channel Estimation and Data Detection in Cell-Free Massive MIMO. IEEE Open J. Signal Process..

[24] Demir Ö.T., Björnson E., Sanguinetti L. (2021). Foundations of User-Centric Cell-Free Massive MIMO.

[25] Gesbert D., Hanly S., Huang H., Shitz S.S., Simeone O., Yu W. (2010). Multi-cell MIMO cooperative networks: A new look at interference. IEEE J. Sel. Areas Commun..

[26] Björnson E., Sanguinetti L. Cell-Free versus Cellular Massive MIMO: What Processing is Needed for Cell-Free to Win?. Proceedings of the 20th IEEE International Workshop on Signal Processing Advances in Wireless Communications, SPAWC 2019.

[27] Lopez-Morales M.J., Chen-Hu K., Garcia-Armada A. (2020). Differential data-aided channel estimation for up-link massive SIMO-OFDM. IEEE Open J. Commun. Soc..

[28] Sun W., Liu J. (2018). 2-to-M Coordinated Multipoint-Based Uplink Transmission in Ultra-Dense Cellular Networks. IEEE Trans. Wireless Commun..

[29] Yang K., Zhang J., Zhang X., Wang W. (2016). Edge aware cross-tier base station cooperation in heterogeneous wireless networks with non-uniformly-distributed nodes. IET Commun..

[30] Li F., Sun Q., Chen X., Peng B., Zhang J., Wong K.K. (2024). Cell-Free Massive MIMO Symbiotic Radio for IoT: RIS or BD?. IEEE Trans. Wireless Commun..

[31] Kenneth O., Mwangi E., Konditi D.B.O. (2025). Optimizing UAV location for deployment in Cell-Free Massive MIMO Networks Using a Soft Actor-Critic Reinforcement Learning. IEEE Access.

[32] Van Chien T., Ngo H.Q., Chatzinotas S., Di Renzo M., Ottersten B. (2021). Reconfigurable intelligent surface-assisted cell-free massive MIMO systems over spatially-correlated channels. IEEE Trans. Wireless Commun..

[33] Zhang Q., Zhao J., Zhang R., Yang L. (2024). Downlink Transmissions of UAV-RIS-Assisted Cell-Free Massive MIMO Systems: Location and Trajectory Optimization. Sensors.

[34] Wu Q., Zhang R. (2020). Towards Smart and Reconfigurable Environment: Intelligent Reflecting Surface Aided Wireless Network. IEEE Commun. Mag..

[35] Zeng Y., Wu Q., Zhang R. (2019). Accessing from the sky: A tutorial on UAV communications for 5G and beyond. Proc. IEEE.

[36] Proakis J.G., Salehi M. (2001). Digital Communications.

[37] Xiao L., Greenstein L.J., Mandayam N.B., Trappe W. (2009). Channel-based detection of sybil attacks in wireless networks. IEEE Trans. Inf. Forensics Secur..

[38] Simon M.K., Alouini M.S. (2005). Digital Communication over Fading Channels.

[39] Basnayaka D.A., Smith P.J., Martin P.A. (2012). Performance Analysis of Dual-User Macrodiversity MIMO Systems with Linear Receivers in Flat Rayleigh Fading. IEEE Trans. Wireless Commun..

[40] Imhof J.P. (1961). Computing the distribution of quadratic forms in normal variables. Biometrika.

[41] Das A. (2024). New methods for computing the generalized chi-square distribution. arXiv.

[42] Liu J., Kwatra S., Kim J. (1995). An analysis of decision feedback detection of differentially encoded MPSK signals. IEEE Trans. Veh. Technol..

[43] Loyka S., Gagnon F. (2004). Performance analysis of the V-BLAST algorithm: An analytical approach. IEEE Trans. Wireless Commun..

[44] Björnson E., Hoydis J., Sanguinetti L. (2017). Massive MIMO networks: Spectral, energy, and hardware efficiency. Found. Trends Signal Process..

[45] Han S., Tellambura C. A complexity-efficient sphere decoder for MIMO systems. Proceedings of the 2011 IEEE International Conference on Communications (ICC).

[46] Liao J., Zhao J., Gao F., Li G.Y. (2022). Deep learning aided low complex sphere decoding for MIMO detection. IEEE Trans. Commun..

[47] Pan J., Ma W.K., Jaldén J. (2013). MIMO detection by Lagrangian dual maximum-likelihood relaxation: Reinterpreting regularized lattice decoding. IEEE Trans. Signal Process..

[48] Sah A.K., Chaturvedi A.K. (2017). Sequential and global likelihood ascent search-based detection in large MIMO systems. IEEE Trans. Commun..

[49] Ali K.S., Abediseid W., Alouini M.S. Sequential decoders for large MIMO systems. Proceedings of the 2014 12th International Symposium on Modeling and Optimization in Mobile, Ad Hoc, and Wireless Networks (WiOpt).

[50] Loyka S.L. (2001). Channel capacity of MIMO architecture using the exponential correlation matrix. IEEE Commun. Lett..

[51] Özdogan Ö., Björnson E., Larsson E.G. (2019). Massive MIMO with spatially correlated Rician fading channels. IEEE Trans. Commun..

